# CHC22 and CHC17 clathrins have distinct biochemical properties and display differential regulation and function

**DOI:** 10.1074/jbc.M117.816256

**Published:** 2017-11-02

**Authors:** Philip N. Dannhauser, Stéphane M. Camus, Kazuho Sakamoto, L. Amanda Sadacca, Jorge A. Torres, Marine D. Camus, Kit Briant, Stéphane Vassilopoulos, Alice Rothnie, Corinne J. Smith, Frances M. Brodsky

**Affiliations:** From the ‡ Division of Biosciences, University College London, London WC1E 6BT, United Kingdom,; the § Institute of Cell Biology/Electron Microscopy, Centre of Anatomy, Hanover Medical School, 30625 Hanover, Germany,; the ¶ Departments of Bioengineering and Therapeutic Sciences, Pharmaceutical Chemistry, Microbiology and Immunology and The G.W. Hooper Foundation, University of California at San Francisco, San Francisco, California 94143,; the ‖ Department of Pharmacology, Fukushima Medical University School of Medicine, Fukushima, Fukushima 960-1295, Japan,; the ** Institut de Myologie, Paris F-75013, France,; ‡‡ Life & Health Sciences, Aston University, Aston Triangle, Birmingham B4 7ET, United Kingdom, and; the §§ Department of Biological Sciences, Warwick University, Coventry CV4 7AL, United Kingdom

**Keywords:** clathrin, glucose metabolism, glucose transporter type 4 (GLUT4), insulin resistance, membrane trafficking, protein self-assembly, secretion, CHC22 isoform, coated vesicles, uncoating ATPase

## Abstract

Clathrins are cytoplasmic proteins that play essential roles in endocytosis and other membrane traffic pathways. Upon recruitment to intracellular membranes, the canonical clathrin triskelion assembles into a polyhedral protein coat that facilitates vesicle formation and captures cargo molecules for transport. The triskelion is formed by trimerization of three clathrin heavy-chain subunits. Most vertebrates have two isoforms of clathrin heavy chains, CHC17 and CHC22, generating two clathrins with distinct cellular functions. CHC17 forms vesicles at the plasma membrane for receptor-mediated endocytosis and at the trans-Golgi network for organelle biogenesis. CHC22 plays a key role in intracellular targeting of the insulin-regulated glucose transporter 4 (GLUT4), accumulates at the site of GLUT4 sequestration during insulin resistance, and has also been implicated in neuronal development. Here, we demonstrate that CHC22 and CHC17 share morphological features, in that CHC22 forms a triskelion and latticed vesicle coats. However, cellular CHC22-coated vesicles were distinct from those formed by CHC17. The CHC22 coat was more stable to pH change and was not removed by the enzyme complex that disassembles the CHC17 coat. Moreover, the two clathrins were differentially recruited to membranes by adaptors, and CHC22 did not support vesicle formation or transferrin endocytosis at the plasma membrane in the presence or absence of CHC17. Our findings provide biochemical evidence for separate regulation and distinct functional niches for CHC17 and CHC22 in human cells. Furthermore, the greater stability of the CHC22 coat relative to the CHC17 coat may be relevant to its excessive accumulation with GLUT4 during insulin resistance.

## Introduction

Clathrin is a triskelion-shaped (three-legged) protein that is recruited by adaptor molecules to cellular membranes where it self-assembles into a latticed coat, driving membrane bud formation. Simultaneously, clathrin organizes adaptor molecules, capturing membrane-embedded cargo for sorting into resulting clathrin-coated vesicles (CCVs)^4^ (1, 2). Clathrin heavy-chain (CHC) subunits form a triskelion by trimerization at their C terminus. Most vertebrates (mice being a notable exception) have two CHC isoforms, CHC17 and CHC22, generated by genome duplication during vertebrate evolution (3). CHC17 triskelia mediate the canonical functions associated with clathrin, including receptor-mediated endocytosis and lysosome biogenesis. Bound to the center of CHC17 triskelia are the clathrin light-chain (CLC) subunits that influence assembly and leg rigidity (1, 4, 5). CHC22, which does not naturally bind CLCs, still shares 85% amino acid sequence identity with CHC17 (6). Despite this high degree of homology, CHC22 plays different cellular roles than CHC17 clathrin. In humans, CHC22 is expressed primarily in muscle and fat (7) and appears transiently during neuronal development (8) in contrast to the ubiquitous and chronic expression of CHC17. In muscle and fat, CHC22 is required for formation of the glucose transporter 4 (GLUT4) storage compartment (GSC), from which GLUT4 is released to the cell surface in response to insulin signaling (7, 9). Although mice have a similar insulin response leading to GLUT4 translocation in muscle and fat, CHC22 clathrin participation is a feature of human GSC formation that is absent from murine models. Additionally, CHC22 accumulates at over-loaded GLUT4 compartments that can form during insulin-resistant type 2 diabetes (T2D), suggesting a potential role for CHC22 in pathogenesis (7).

Here, we compare the biochemical, morphological, and functional properties of CHC17 and CHC22 to understand their ability to operate in separate biological niches and gain insight into the properties of assembled CHC22 that might be relevant to human insulin resistance. In this study, we have produced the first images of purified CHC22 CCVs and triskelia, both resembling structures formed by CHC17 (10–12). We demonstrate that CHC22 and CHC17 form segregated coated vesicles and that regulation of their membrane recruitment and disassembly, as well as uncoating, is distinct. Although a previous report suggested that CHC22 could functionally substitute for CHC17 at the plasma membrane (13), we found this not to be the case for either endocytic-coated vesicle formation or transferrin uptake. Thus fundamental biochemical differences underlie the non-redundant functions of the two clathrins. These differences are relevant to CHC22 function in human health and disease, and could account for some of the documented differences in human and murine glucose metabolism noted from mouse models (14, 15).

## Results

### CHC22 forms coated vesicles that are distinct from those formed by CHC17

Functional studies indicate that CHC22 and CHC17 operate in separate membrane traffic pathways in formation of the human GLUT4 storage compartment (7), and the two isoforms are separately immunoprecipitated from cell lysate (6). Our previous immunofluorescence studies in human skeletal muscle showed that CHC17 and CHC22 are typically found at separate locations in cells, with about 15% overlap (by Pearson's correlation) (7). This suggests that a small fraction of intracellular membrane might be coated with mixtures of CHC17 and CHC22. However, CHC22 was implicated as a component of CHC17-coated vesicles by proteomics experiments, which showed that CHC22 was depleted from CCV preparations following siRNA-mediated down-regulation of CHC17 (16). We therefore sought to establish the degree to which CHC22 and CHC17 form mixed lattices in cells.

**Figure 1.**
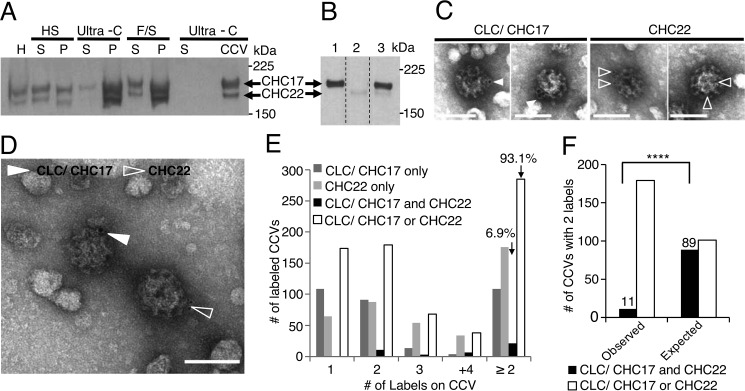
**CHC22 forms a latticed coat on vesicles that are distinct from CHC17-coated vesicles.** *A,* immunoblot of CCV purification from HeLa cell homogenate (*H*) showing the supernatant (*S*) and pellet (*P*) fractions for each differential centrifugation step and the final CCV pellet (*HS,* high-speed; *Ultra-C*, ultra-centrifugation; *F*/*S,* high-speed centrifugation in Ficoll/sucrose). Samples were analyzed by TAE SDS-PAGE to separate the two CHC isoforms, which were detected with a mixture of anti-CHC17 (TD.1) and anti-CHC22 (SHL-KS) antibodies followed by HRP-conjugated secondary antibodies. *B,* HeLa cell lysates were separated by TAE SDS-PAGE, transferred to nitrocellulose, and analyzed by immunoblotting. Blot lanes were cut into strips and incubated separately with anti-CHC17 (TD.1, *lane 1*), anti-CHC22 (SHL-KS, *lane 2*), or mixed TD.1 and SHL-KS (*lane 3*). Blotted strips were re-joined for imaging. Note that the antibody mixture in *A* was optimized to detect both CHC isoforms equally (“Experimental procedures”), whereas the mixture in *B* represents the antibody dilutions used in the individually blotted strips. *C,* CCV fraction from *A* was co-labeled with immunogold for CLC bound to CHC17 (CLC/CHC17, 10 nm particles, *filled arrowheads*) and CHC22 (5 nm particles, *open arrowheads*) and stained with uranyl acetate. Individual vesicles labeled for CLC/CHC17 or CHC22 are shown. Brightness of the images was adjusted to improve visibility of gold particles. *D,* larger image from co-labeling as in *C* showing vesicles labeled independently for CLC/CHC17 and CHC22 in the same field, as for *C. E,* for CCVs labeled with 1, 2, 3, or ≥4 gold particles, the number labeled individually for each CHC isoform or labeled for both isoforms is shown. For the total number of CCVs (*n* = 306) labeled with two or more gold particles (≥2), the percentage labeled for both CHC isoforms (21 total), or only one isoform (285 total) is indicated. *F,* observed numbers of CCVs decorated with two gold particles (from *E*) that are labeled for both isoforms or labeled individually compared with the expected labeling if coats comprise both isoforms (accounting for labeling efficiency and relative isoform abundance, see “Experimental procedures”). Eleven observed dual-labeled CCVs was significantly lower than the expected 89 dual-labeled CCVs if mixed coats (χ^2^ test, **** = *p* < 0.0001, *n* = 190 CCVs with two labels, df = 1; “Experimental procedures”). For gels and blots, the migration positions of molecular mass markers are indicated at the *right* in kilodaltons. *Scale bars* (*C* and *D*) = 100 nm.

To this end, we purified clathrin-coated vesicles from HeLa cells using the same CCV purification protocol as in the cited proteomics study (16, 17), and note that HeLa cells (atypical for their tissue origin) express CHC22 at similar levels to human myotubes (9). We found that a Tris/acetate/EDTA (TAE)-buffered SDS-PAGE system described previously (18) separates CHC22 and CHC17 by electrophoretic mobility (Fig. 1, *A* and *B*) and this analysis revealed that both isoforms were present in the coated vesicle preparation (Fig. 1*A*), suggesting that CHC22 is found in structures of a similar size and density as CHC17 structures known to be CCVs. We then examined the CCVs by immunoelectron microscopy using an anti-CLC antibody to detect CHC17 and an isoform-specific anti-heavy-chain antibody to detect CHC22. Immunogold labeling with secondary antibodies coupled to different-sized gold particles revealed that CCVs were primarily labeled for only one CHC isoform (Fig. 1, *C* and *D*). Quantification showed that 93.1% of CCVs with two or more gold particles were labeled by antibodies recognizing either CHC22 or CHC17 but not both (Fig. 1*E*). Statistical analysis of CCVs labeled with two gold particles demonstrated that the number of CCVs co-labeled for both CHC17 and CHC22 was significantly below the number that would be expected to co-label for both clathrins if mixed coats are formed, taking into account the efficiency of antibody labeling and the relative abundance (9) of the two clathrin heavy chains in HeLa cells (Fig. 1*F*; “Experimental procedures”). Negatively stained images showed that vesicles labeled only for CHC22 were coated with lattices visually similar to CHC17 lattices (Fig. 1, *C* and *D*). This analysis by electron microscopy reveals that when purified from cells, CHC22 CCVs are predominantly distinct from those formed by CHC17 (Fig. 1), although their lattices share morphology.

### CHC22 and CHC17 coats have distinct biochemical dissociation properties and both clathrins are triskelia

**Figure 2.**
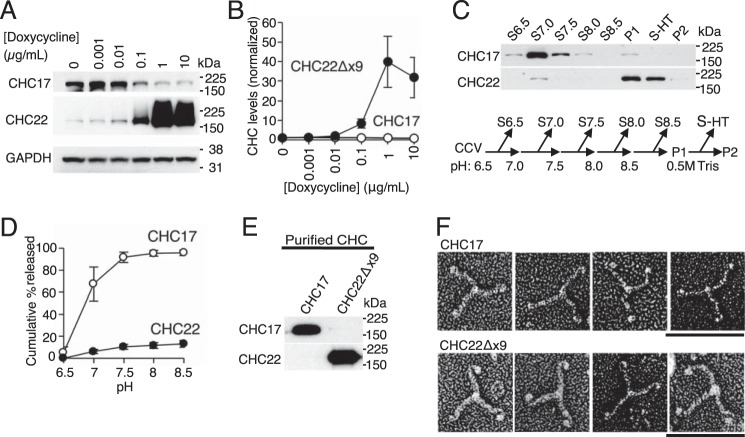
**The CHC22 clathrin coat is resistant to pH-dependent stripping from membranes and CHC22Δx9 is a triskelion.** *A* and *B,* HeLa-CHC22Δx9-TO cells were incubated with doxycycline for 24 h at the concentrations indicated and cell lysates were prepared. Representative immunoblot (*A*) and quantification (*B*, average ± S.E.) of signals for CHC17 and CHC22Δx9 relative to no doxycycline treatment, normalized to GAPDH for each sample (*n* = 5). *C,* purified CCVs from untransfected HeLa cells were exposed to increasing pH by sequential suspension in buffer with the indicated pH and subsequent centrifugation (see flow scheme below). Representative immunoblot (above) of CHC isoforms released from CCVs in the indicated supernatants (*S*) designated for each pH treatment; *P1*, final CCV pellet after sequential treatment with increasing pH; *S-HT*, supernatant released following treatment of P1 with high Tris (0.5 m); *P2*, CCV pellet after high Tris treatment. *D,* quantification of the cumulative percent released after increasing pH treatment, as in *C* (*n* = 3). *E,* immunoblot analysis of CHCs purified by differential stripping of CCVs isolated from untransfected HeLa (CHC17) and doxycycline-treated HeLa-CHC22Δx9-TO cells. *F,* purified CHC isoforms from *E* visualized by deep-etch electron microscopy. Representative electron micrographs of CHC17 (*upper panels*) and CHC22Δx9 (*lower panels*). Brightness and contrast of the images were adjusted to improve visibility of the triskelia. *Scale bar* = 50 nm. For all blots, the migration positions of molecular mass markers are indicated at the *right* in kilodaltons and the specificity of the antibody used for blotting is shown at the *left*.

The clathrin coat was first described for its lattice (clathrate) structure (19), and later CHC17 clathrin was shown to be a triskelion (10–12). We previously showed by size-exclusion chromatography that the recombinantly expressed C-terminal third of CHC22 (Hub fragment, residues 1074–1640) forms a trimer (6). However, due to low solubility (different from the CHC17 Hub), it was not possible to obtain sufficient CHC22 Hub protein to visualize this fragment. Without a human tissue source for purifying CHC22, we addressed whether we could purify CHC22 following over-expression in tissue culture and sought to take advantage of an existing HeLa cell transfectant (HeLa-CHC22Δx9-TO) that can over-express CHC22Δx9 under the control of a Tet-On promoter (6). CHC22Δx9 protein lacks 60 amino acids (457–507 encoded by Exon 9) comprising the CHCR0 region between the distal leg and the N-terminal domain, so that CHC22Δx9 is virtually full-length (1580 of 1640 residues) with intact trimerization, proximal, and distal leg and terminal domains. Doxycycline treatment of HeLa-CHC22Δx9-TO caused CHC22Δx9 to be the dominant clathrin in these cells (Fig. 2, *A* and *B*). We then addressed whether CHC22 and CHC17 can be differentially dissociated from HeLa CCVs. It was previously observed that high pH combined with low salt concentrations efficiently strips CHC17 from membranes (20), so we tested the effects of pH change in low salt on releasing CHC22 from CCVs purified from untransfected HeLa cells. Sequential exposure of mixed purified CCVs to increasing pH removed CHC17 without substantially dissociating CHC22 from their respective CCVs. Only 13% of CHC22 was solubilized at pH 8.5, whereas 95% of CHC17 was released from the CCV mixture (Fig. 2, *C* and *D*). From the pellet of CCVs exposed to pH 8.5, we were able to solubilize CHC22 by exposure to 0.5 m Tris buffer, another method used in standard protocols for purifying CHC17 (21) (Fig. 2*C*).

To obtain purified CHC22Δx9, CCVs were isolated from HeLa-CHC22Δx9-TO cells treated with doxycycline, then exposed to increasing pH to remove CHC17, followed by Tris stripping the final pellet to release CHC22Δx9. To obtain purified CHC17 for comparison, CCVs were purified from untransfected HeLa cells (with a natural 10-fold excess of CHC17 over CHC22), then exposed to pH 7.0, releasing CHC17 and leaving CHC22 behind on pelleted membranes. Immunoblotting with isoform-specific antibodies confirmed the purity of the CHC22Δx9 and CHC17 obtained by these methods (Fig. 2*E*). Analysis of the separated purified clathrin isoforms by deep-etch electron microscopy showed that CHC22Δx9 has the same triskelion configuration and approximate dimensions as CHC17 (Fig. 2*F*), providing morphological evidence that CHC22 is a triskelion.

### Differential enzymatic uncoating of CHC22 and CHC17

**Figure 3.**
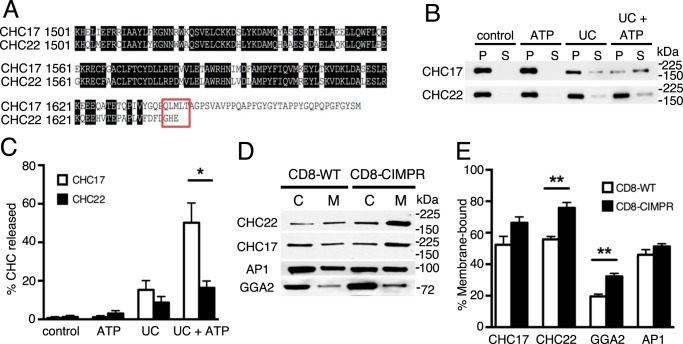
**CCV uncoating and membrane recruitment differ for CHC17 and CHC22.** *A,* sequence alignment of the C-terminal portions of human CHC17 and CHC22. Identical amino acids are marked in *black*. The Hsc70 recognition site at the C-terminal end of CHC17 (24) is boxed in *red*, showing the difference from CHC22. *B,* CCVs from HeLa cells were incubated without (control) or with the UC (Hsc70 and cofactor auxilin) plus or minus ATP. Uncoated clathrin triskelia were separated from residual CCVs by centrifugation. CHC17 or CHC22 were detected in resulting supernatants (*S*) and pellets (*P*) by separately immunoblotting each sample with isoform-specific antibodies. *C,* quantification of uncoating efficiency (release of CHCs into supernatant, S/(S+P) signals) from *n* = 4 experiments as in *B*. *, *p* < 0.05 by Student's *t* test, CHC17 *versus* CHC22. *D,* representative immunoblot showing CHC22, CHC17, AP-1, and GGA2 in cytosolic (*C*) or membrane (*M*) fractions from HeLa cells stably expressing wild-type CD8 (CD8-WT) or chimeric CD8 with the intracellular domain of CI-MPR (CD8-CIMPR). *E,* quantification of *n* = 4–5 experiments as in *C*, expressed as membrane fraction signal divided by total signal, M/(C + M). **, *p* < 0.01 by Student's *t* test, CD8-CIMPR *versus* CD8-WT. For all blots, the migration positions of molecular mass markers are indicated at the *right* in kilodaltons and the specificity of the detecting antibodies is indicated at the *left*.

The CHC17 triskelia cycle from the cytosol to nascent CCVs at the plasma membrane or other organelles, and are returned back to the cytosol by uncoating fully closed CCVs (1). Uncoating is mediated by an ATPase complex consisting of the 70-kDa heat-shock cognate protein (Hsc70) and auxilin, and requires ATP hydrolysis (22, 23). Notably, CHC22 lacks the 5-residue recognition site (QLMLT) for Hsc70 binding to CHC17 (24) (Fig. 3*A*). Our previous work showed that, at steady state, most cellular CHC22 is associated with membranes, whereas CHC17 is about half-cytosolic at any one time (6), suggesting that CHC22 disassembly dynamics are different from those of CHC17. Furthermore, the *in vitro* conditions necessary to dissociate CHC22 from CCVs for purification suggested differences in properties of CHC22 lattices compared with CHC17 lattices. We therefore addressed whether CHC22 CCVs are uncoated by the same cellular mechanism that operates for CHC17 coat disassembly. CCVs purified from HeLa cells were incubated with recombinant uncoating complex (UC, Hsc70 plus a functional fragment of auxilin) with and without ATP. After centrifugation, CHCs released to the supernatant or remaining in the pellet were assessed by immunoblotting with isoform-specific antibodies (Fig. 3*B*). The UC plus ATP effectively removed more than 50% of the CHC17 from CCVs under these conditions, but only 16% of CHC22 was released (Fig. 3*C*). These data show that under conditions where CHC17 coats are efficiently removed, the Hsc70–auxilin complex is significantly less efficient in uncoating CHC22 assemblies, consistent with loss of Hsc70 efficacy when the QLMLT sequence is deleted from CHC17 (24). These findings indicate that CHC22 uncoating proceeds differently from CHC17 uncoating. Cellular dissociation of CHC22 coats may be performed by different proteins or under different conditions than cellular CHC17 disassembly.

### Differential membrane recruitment of CHC22 and CHC17

Clathrin recruitment to membranes is driven by adaptor proteins that recognize phospholipids at specific intracellular locations and selectively interact with cargo (25). Immunoisolation of CHC22 from HeLa cells and human skeletal muscle in earlier studies showed co-purification of the adaptors AP1, AP3, and GGA2 (Golgi-associated, γ adaptin ear-containing, adenosine diphosphate ribosylation factor–binding protein 2), but not the plasma membrane-associated adaptor AP2 (6, 7). Although the adaptors bound to CHC22 also bind to CHC17, immunoisolation showed more GGA2 co-isolated with CHC22 than with CHC17 relative to the other adaptors (7), suggesting a possible mechanism for their differential recruitment. To address CHC interactions with GGA2, we examined whether forced membrane association of GGA2 could attract CHC22 and/or CHC17 to membranes. It has been shown that membrane association of GGA2 dramatically increases in HeLa cells stably expressing a chimeric CD8 protein with the cytoplasmic tail of CIMPR (CD8-CIMPR) (26). In these cells, we assessed the proportion of adaptors and clathrin that were membrane associated by immunoblotting the membrane and cytosol fractions (Fig. 3, *D* and *E*). Compared with cells expressing CD8 without the cytoplasmic domain of CIMPR (CD8-WT), the fractions of GGA2 and CHC22 associated with membranes were significantly increased in the CD8-CIMPR cells. The membrane localization of CHC17 appeared slightly increased in the CD8-CIMPR cells, but this was not statistically significant (Fig. 3*E*). Thus, increased membrane association of GGA2 correlates with greater recruitment of CHC22 than CHC17, supporting the biochemical evidence that preferential adaptor binding properties contribute to differential localization and functions of CHC22 and CHC17.

### CHC22 does not substitute for CHC17 in endocytosis

Our previous work localized CHC22 function to internal membrane traffic and showed by fluorescence microscopy that down-regulation of CHC22 did not affect receptor-mediated endocytosis of fluorescent transferrin (Tf) or epidermal growth factor (7, 9), consistent with lack of AP2 binding or co-localization with AP2 (6, 7). However, it was reported that in cells treated with siRNA to down-regulate CHC17, over-expressed CHC22 was apparently able to rescue endocytosis of transferrin, suggesting potential redundant roles of the two heavy-chain isoforms (13).

**Figure 4.**
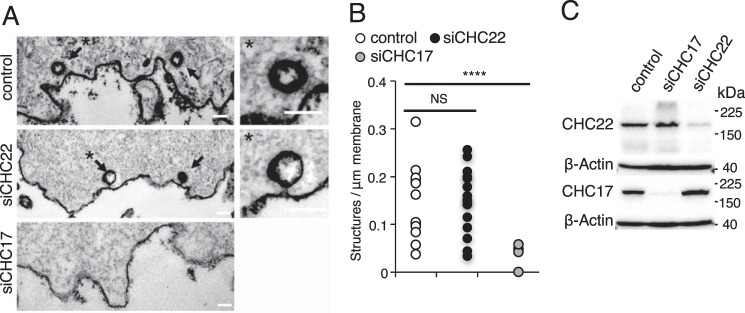
**Clathrin-coated structures at the plasma membrane are eliminated by CHC17 depletion but not affected by CHC22 depletion.** *A,* representative electron micrographs illustrate closed clathrin-coated structures proximal to the plasma membrane of HeLa cells treated with control siRNA, siRNA targeting CHC22 (*siCHC22*), or siRNA targeting CHC17 (*siCHC17*). *Arrows,* clathrin-coated structures; *, structures at the *left enlarged at the right* to show clathrin coat. *Scale bars* = 100 nm. *B,* quantification of clathrin-coated structures (*open* (*pits*) and *closed*) proximal to the plasma membrane in HeLa cells treated with siRNA as in *A*. Clathrin-coated structures per micrometer of plasma membrane are reduced in siCHC17-treated cells *versus* control (****, *p* < 0.0001), siCHC22-treated cells do not differ significantly from control cells (*NS*, not significant at *p* < 0.05); *n* = 15 cells per condition (Student's *t* test). *C,* immunoblot of HeLa cells in *A* and *B* showing efficient depletion of CHC17 and CHC22 by siRNA treatment; β-Actin shown as loading control. The migration positions of molecular mass markers are indicated at the *right* in kilodaltons and the specificity of the detecting antibody is indicated at the *left*.

To address whether CHC22 forms coated vesicles at the plasma membrane, we used electron microscopy to quantify the presence of clathrin-coated structures at the plasma membrane in cells treated with siRNA targeting CHC22 or CHC17 (Fig. 4). We found, as expected, that siRNA-mediated depletion of CHC17 caused near-complete elimination of clathrin-coated structures at the plasma membrane compared with control siRNA-treated cells. However, CHC22 depletion had no significant effect on the prevalence of clathrin-coated pits or vesicles at the plasma membrane compared with cells treated with control siRNA (Fig. 4). Together with our previous work showing that CHC22 is not localized to the plasma membrane (6, 9), these new data establish that endogenous CHC22 does not support formation of clathrin-coated structures at the plasma membrane.

**Figure 5.**
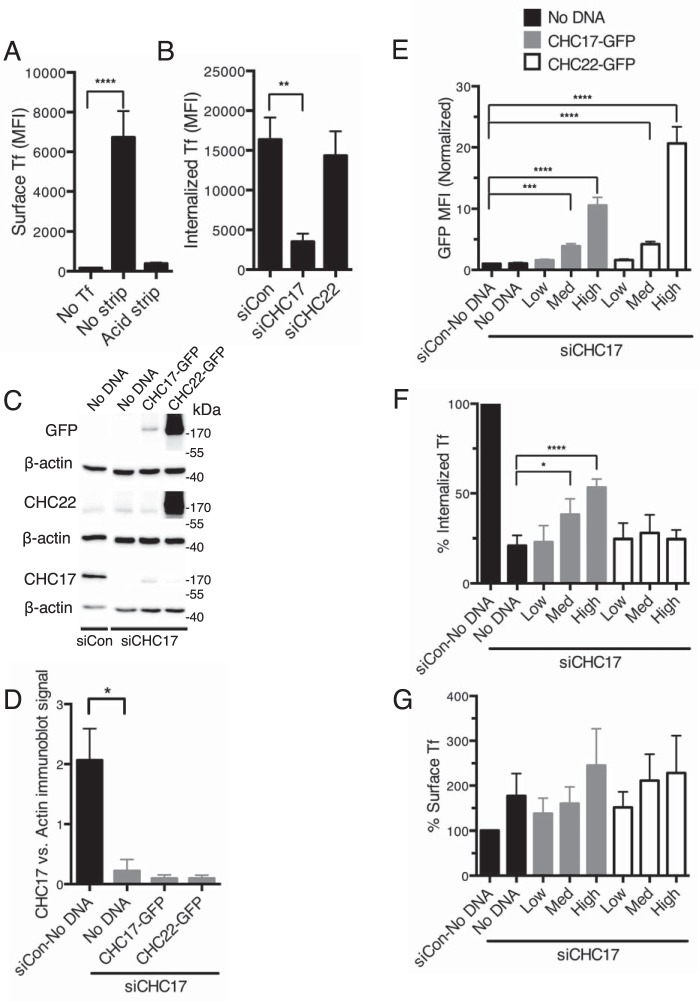
**Receptor-mediated endocytosis of transferrin is not rescued by CHC22 overexpression in the absence of CHC17.** *A,* control conditions for FACS quantification of fluorescent Tf uptake. Basal fluorescence and maximal surface fluorescence were determined from cells incubated with Tf-AF647 or not (*No Tf*), kept on ice to block endocytosis. The efficiency of surface Tf stripping was assessed by comparing the fluorescence of unstripped cells (*No strip*) to cells with bound Tf exposed to an acid wash (*Acid strip*). The mean fluorescence intensity (*MFI*) of bound Tf-A647 is shown. (One-way ANOVA, no Tf *versus* no strip and acid strip conditions, ****, *p* < 0.0001, *n* = 6–18.) *B,* quantification of Tf uptake in cells treated with control siRNA (*siCon*) or with siRNA targeting CHC17 (*siCHC17*) or CHC22 (*siCHC22*). The MFI of the internalized Tf-A647 (after surface stripping) is shown. (One-way ANOVA, siCon *versus* siCHC17 and siCHC22 conditions, **, *p* < 0.01, *n* = 6.) *C,* representative immunoblots showing endogenous CHC17 and CHC22 and expression of the GFP-tagged CHC17 and CHC22 rescue constructs in HeLa cells transfected with siCon or siCHC17, used for experiments in *B* and *E-G*. For all blots, the migration positions of molecular mass markers are indicated at the *right* in kilodaltons (*kDa*) and the specificity of the blotting antibody is shown at the *left*. Note that the TD.1 antibody used to detect CHC17 does not detect GFP-CHC17 due to epitope masking by the tag. The faint band of the size of the endogenous CHC17 that is visible for the siCHC17 + GFP-CHC17 condition represents a cleavage product missing the GFP. The double band detected by the anti-CHC22 antibody represents full-length transfected GFP-CHC22 and a cleavage product missing the GFP plus endogenous CHC22. *D,* quantification of CHC17 immunoblot signals from the depletion experiments were analyzed in *B* and *E-G* (Student's *t* test, siCon *versus* siCHC17 (no DNA), *, *p* < 0.05, *n* = 3), normalized to actin immunoblot signals for each sample. *E,* FACS-based quantification of GFP-CHC17 and GFP-CHC22 expression in HeLa cells after treatment with siCHC17 or siCon. The GFP-positive population of cells was divided in three groups with low, medium, and high MFI of the transfected GFP-CHC and for each group, the MFI for fluorescent Tf-AF647 was normalized to the Tf-AF647 MFI of the *siCon*, no DNA condition for panels (*E-G*; one-way ANOVA, control (*siCon*, no DNA) *versus* all siCHC17 conditions, ****, *p* < 0.0001; ***, *p* < 0.001, *n* = 6). *F,* Tf-AF647 uptake measured by FACS for cells in *C-G*. Results were normalized to control (siCon, no DNA) and expressed as percent internalized Tf (one-way ANOVA, siCon *versus* all siCHC17 conditions, ****, *p* < 0.0001; *, *p* < 0.05, *n* = 6). *G,* surface-bound Tf-AF647 prior to uptake measured by FACS for assays in *B-G* (one-way ANOVA, control (*siCon*, no DNA) *versus* all siCHC17 conditions, not statistically significant, *p* > 0.05).

To address whether CHC22 can substitute for CHC17 functionally in endocytosis, we used a fluorescence-activated cell sorter (FACS) to quantitatively measure Tf uptake and directly measure levels of expression of each clathrin. To ensure analysis of true receptor-mediated endocytosis and not pinocytosis, we exposed cells to 10 μg/ml of fluorescently labeled Tf at 4 °C for surface binding, then washed to remove excess (unbound) Tf prior to warming the cells to 37 °C. After uptake, residual surface Tf was stripped with acidic buffer (Fig. 5*A*) so that cell-associated fluorescence represented only internalized Tf from the pulsed exposure (“Experimental procedures”). To establish whether CHC22 can replace CHC17 function in receptor-mediated Tf endocytosis, we confirmed that down-regulation of CHC17 but not CHC22 affected Tf uptake (Fig. 5, *B-D*). Cells treated to down-regulate either CHC17 or CHC22 were then sequentially transfected to express siRNA-resistant CHC17 or CHC22, tagged with GFP (Fig. 5, *C-G,* supplemental Fig. S1). The GFP tag on both “rescue” constructs made it possible to measure their relative expression levels (Fig. 5*E*), so that we could assess whether CHC22 expressed at equivalent (or higher) levels to CHC17 can rescue endocytosis as effectively. We observed that in the absence of CHC17, transfection of CHC22 did not rescue Tf uptake in this assay (Fig. 5*F*), even when expressed at a higher level than that effective for rescue by CHC17 (Fig. 5*E*). Starting levels of surface-bound Tf were similar for CHC17-depleted cells transfected with either rescue construct (Fig. 5*G*). Transfection of HeLa cells that were treated with control siRNA or with siRNA targeting CHC22 confirmed that internalization was not reduced by these treatments and showed increased Tf uptake when GFP-CHC17 was transfected but not when GFP-CHC22 was transfected (supplemental Fig. S1).

Taken together, the electron microscopy analysis of coated pit formation and the Tf uptake assays presented here are consistent with our previous studies, and strongly support the conclusion that CHC22 does not participate in receptor-mediated endocytosis at the plasma membrane.

## Discussion

In this study, we examined the morphology and biochemical properties of CHC22 clathrin, which we have previously shown is involved in regulation of GLUT4 membrane traffic in humans (7). We show for the first time that CHC22 forms a latticed coat on CCVs that are distinct from CHC17-coated vesicles. We also extend our initial biochemical observations that the C-terminal third of CHC22 forms a trimer (6) to show that the nearly full-length CHC22Δx9, and by inference full-length CHC22, has a triskelion shape similar to that of CHC17. Notably, we find that the CHC22 lattice is stable under alkalinizing conditions that disassemble CHC17 and that its uncoating properties differ from those of CHC17. Specifically, the uncoating ATPase complex of Hsc70 and auxilin that acts to remove CHC17 from membranes does not effectively uncoat CHC22 under the same conditions, suggesting that CHC22 is uncoated less efficiently in cells or uncoated by an alternative mechanism. Finally, we show that CHC22 does not function in coat formation at the plasma membrane or receptor-mediated endocytosis of transferrin and that the “internal” adaptor GGA2 plays a role in preferential recruitment of CHC22 to membranes over CHC17. Together, our data indicate that different biochemical properties underlie the different cellular functions of the two vertebrate CHCs.

Human GLUT4 trafficking epitomizes the different cellular functions of CHC22 and CHC17. In humans, 70% of post-prandial blood glucose is cleared by import into skeletal muscle and fat using the GLUT4 transporter that is released from its intracellular storage compartment in response to insulin (27, 28). Our previous work supports a role for CHC22 in targeting GLUT4 to this insulin-responsive compartment, whereas CHC17 is known to control a different part of the GLUT4 trafficking pathway by mediating endocytosis of GLUT4 to terminate insulin-stimulated glucose uptake (7, 29). In muscle from insulin-resistant T2D patients, GLUT4 trafficking is perturbed such that GLUT4 is not released to the cell surface in response to insulin. Instead, GLUT4 intracellular storage compartments become over-loaded with GLUT4. CHC22 clathrin accumulates at the abnormal GLUT4 compartments, whereas CHC17 localization seems unaffected (7). We identify GGA2 as a preferential CHC22 adaptor, and GGA2 has been previously shown to mediate trafficking of GLUT4 to the same intracellular GLUT4 storage compartment where CHC22 accumulates (30). Furthermore, we demonstrate here that CHC22 lattices are resistant to cellular and biochemical uncoating conditions that disassemble CHC17, indicating that CHC22 coats are apparently more stable and could be pathogenic in excess. Indeed, we showed previously that expression of CHC22 in mouse muscle, where it is not normally expressed, excessively traps GLUT4 in intracellular compartments leading to elevated blood glucose (7). Together these observations lead us to speculate that enhanced CHC22 accumulation at the aberrant GLUT4 storage compartment that results from insulin resistance might contribute to and exacerbate GLUT4 retention. It is notable that separate roles for CHC22 and CHC17 were also observed in a cell line model of transient CHC22 expression during neuronal development (8). Thus, distinct roles of these clathrins have potential significance for additional physiological pathways.

The findings reported here further support segregated intracellular functions of CHC22 and CHC17 clathrin and resolve debates about their functional redundancy (13, 16). We demonstrate by electron microscopy that CHC22 does not form endocytic structures at the plasma membrane, when present at its normal levels of expression, even when CHC17 is down-regulated. Furthermore, consistent with lack of CHC22 co-immunoprecipitation with the AP2 adaptor (6, 7), we show that when CHC17 is depleted from cells to inhibit receptor-mediated endocytosis of Tf, expression of CHC22 does not rescue CHC17 function. We designed this latter experiment to specifically test receptor-mediated endocytosis because previous experiments reporting the ability of CHC22 to replace depleted CHC17 in transferrin uptake (8, 13) used a 5- or 10-fold higher concentration of transferrin without washing out excess prior to uptake or stripping residual surface-bound Tf after uptake. In these previous studies, internalization was assessed by confocal microscopy and receptor-mediated endocytosis would have been accompanied by pinocytosis of unbound Tf. In the present study, we used quantitative FACS, non-saturating concentrations of transferrin, and washed away excess before initiating internalization and residual surface bound Tf was removed, thus measuring only internalized Tf. In this assay, CHC22 did not restore Tf uptake that was inhibited by depletion of CHC17, even though CHC22 expression was higher than levels of CHC17 that rescued Tf endocytosis. Here we further support the non-redundant functions of the two clathrins by our observation that CHC22 and CHC17 form separate CCVs in cells. We also did not observe decreased CHC22 upon CHC17 depletion (Figs. 4*C* and 5*C*), as suggested by Borner *et al.* (16). Using siRNA targeting the separate isoforms we often see the opposite effect, that CHC22 levels tend to increase upon CHC17 depletion (7, 9) (although no significant increase was observed in the experiments shown here). We have shown that whereas CHC22 does not co-immunoprecipitate with AP2, it does associate with some adaptors that are also bound by CHC17, including AP1 and GGA2 (6, 7). If CHC17 is depleted, sites on these adaptors would become available for CHC22 binding, possibly explaining increased stability of CHC22 through increased membrane association upon CHC17 loss. Here we identify GGA2 as a shared adaptor that seems to preferentially recruit CHC22 to intracellular membranes. Because this preference is not dramatic, we propose that there are additional adaptors unique to CHC22 recruitment, which would be required to increase local CHC22 concentration such that it could compete with CHC17 for shared adaptor interactions.

In conclusion, we find that although CHC22 is similar to CHC17 in morphology, the biochemical characteristics of these two clathrin heavy chains with 85% sequence identity are quite different. Their uncoating and membrane recruitment are differentially regulated, and we propose that elucidation of these mechanisms could provide insight into the pathogenesis and treatment of insulin resistance leading to T2D. Furthermore, identification of CHC22's triskelion shape and its ability to form stable lattices set the stage for further structural and biochemical understanding of CHC22 regulation and its role in human glucose homeostasis (7), as well as in pathways of neuronal development (8).

## Experimental procedures

### Cell culture

HeLa cells (Subtype 229) were grown in DMEM high glucose supplemented with 10% fetal bovine serum (FBS), 50 units/ml of penicillin, and 50 μg/ml of streptomycin. HeLa cells stably expressing a doxycycline-inducible construct, pJM601-CHC22Δx9 (HeLa-CHC22Δx9-TO cells) (6) were supplemented with 50 μg/ml of G418 and grown in media supplemented with tetracycline-free FBS. Expression of CHC22Δx9 was induced in HeLa-CHC22Δx9-TO cells by adding 1 μg/ml of doxycycline (or as indicated), and cells were cultured 24 h before harvest. Stable HeLa cell clones expressing CD8 constructs (from Matthew Seaman, Cambridge University) were produced and cultured as described (26).

### siRNA-mediated depletion of CHC isoforms

HeLa cells were transfected with siRNAs targeting CHC17 (40 nm), CHC22 (20 nm), or All-Star Negative control siRNA (40 nm) (Qiagen) (31) using JetPrime® (Polyplus) transfection reagent according to the manufacturers' instructions. Cells were analyzed 72 or 96 h after transfection was initiated.

### Preparation of membrane and CCV fractions

CCV preparation was similar to Hirst *et al.* (17). HeLa cells or HeLa-CHC22Δx9-TO cells exposed to 1 μg/ml of doxycycline were grown on four 500-cm^2^ dishes. Cells were rinsed with ice-cold PBS followed by ice-cold Buffer A (0.1 m MES, pH 6.5, 0.2 mm EGTA, 0.5 mm MgCl_2_, 0.02% NaN_3_, 0.2 mm PMSF), then scraped in 4 ml of Buffer A per dish. Homogenization by Potter-Elvehjem homogenizer (15 strokes) or by sonication was followed by centrifugation (2,000 × *g* for 30 min), and supernatants were treated with 10 μg/ml of RNase A (30 min, 4 °C). Membranes were then pelleted (109,000 × *g*, 30 min) and resuspended in 300 μl of Buffer A. To separate CCVs from the membrane fraction, the suspension was mixed with equal volumes (300 μl) of 12.5% Ficoll and 12.5% sucrose in Buffer A and centrifuged (17,400 × *g*, 30 min). The supernatant was diluted with 4 volumes of Buffer A (2.4 ml), and CCVs were pelleted (109,000 × *g*, 30 min). Pellets were resuspended in 100 μl of Buffer A.

### Isolation of CHC isoforms for imaging

To purify CHC22Δx9, CCV pellets isolated from HeLa-CHC22Δx9-TO cells suspended in HEPES buffer (10 mm HEPES-NaOH, pH 6.5, 0.2 mm EGTA, 0.02% NaN_3_), then centrifuged (100,000 × *g*, 30 min). The resulting pellet was stripped gradually of CHC17 by sequential exposure to HEPES buffer with increasing pH (10 mm HEPES-NaOH, pH 7.0, 7.5, 8.0, 8.5, 0.2 mm EGTA, 0.02% NaN_3_), spinning out the pellet after each extraction. The final pellet was resuspended in high Tris buffer (50% Buffer A and 50% 1 m Tris-HCl, 2 mm EDTA, 0.4 mm PMSF, pH 7.0), then centrifuged to yield a supernatant containing pure CHC22Δx9, which was imaged. To produce a similar preparation of purified CHC17, CCVs were isolated from untransfected HeLa cells and washed in HEPES buffer, pH 6.5, then CHC17 was stripped from the resulting pellet by extraction in HEPES buffer, pH 7.0. Deep-etch replicas of each separated CHC were prepared by adsorbing proteins on mica flakes and then quick-freezing, freeze-fracturing, deep-etching, and platinum-replicating (32), in a fee-for-service arrangement at the Washington University Center for Cellular Imaging, St. Louis, MO.

### Separation of CHC isoforms by SDS-PAGE

CHC isoforms were separated by electrophoresis in continuous TAE-buffered SDS-PAGE (18). TAE gels were prepared with 6.3% acrylamide, 0.17% bisacrylamide, 0.1 m Tris-HCl, 0.2 m sodium acetate, 0.02 m EDTA, 0.1% SDS, pH 7.4, and polymerized with 0.075% tetramethylethylenediamine and 0.75% ammonium persulfate. Samples in standard Laemmli SDS buffer were electrophoresed 3 h in 0.1 m Tris-HCl, 0.2 m sodium acetate, 0.02 m EDTA, 0.1% SDS, pH 7.4, at 100 mA (∼70 V), at 22 °C.

### Immunoblotting

Protein samples were separated by SDS-PAGE (10% acrylamide or TAE, 6.3% acrylamide) and transferred to nitrocellulose. TAE gels were incubated in transfer buffer (50 mm Tris, 39 mm glycine, 0.037% SDS, 20% methanol pH 8.3) for 30 min at 22 °C prior to transfer. Membranes were labeled with primary antibodies (1–5 μg/ml) as follows: anti-CHC17 (TD.1, self-raised mouse monoclonal against CHC17 terminal domain) (33) or anti-CHC22 (SHL-KS, affinity purified self-raised rabbit polyclonal against CHC22 C terminus cross-absorbed against the CHC17 C terminus) (6, 34). For Fig. 1*A*, TD.1 antibody at 0.27 μg/ml was mixed with SHL-KS to achieve equivalent labeling of both CHC isoforms. Horseradish peroxidase (HRP)-conjugated secondary antibodies were detected using ECL^TM^ reagent (Amersham Biosciences). Quantification was performed using ImageJ (NIH).

### Uncoating assays

Uncoating experiments were modified from a published protocol (35). To prepare CCVs, HeLa cells (confluent 500 cm^2^ dish) were rinsed three times in PBS (4 °C), removed by scraping, resuspended in 900 μl of uncoating buffer (40 mm HEPES, pH 7.4, 75 mm KCl, 4.5 mm MgCl_2_, 1.5 mm CaCl_2_) supplemented with protease inhibitors (2 μg/ml of aprotinin, 1 mg/ml of leupeptin, 0.005% PMSF), and homogenized by a Potter-Elvehjem homogenizer (5 strokes). Homogenates were centrifuged (1000 × *g*, 15 min), then the post-nuclear supernatants were centrifuged (10 min, 100,000 × *g*). To generate a crude CCV fraction, the pellet was exposed to detergent to remove uncoated membranes, then rinsed to remove excess detergent, resuspended in 900 μl of uncoating buffer (room temperature, 15 min to release free triskelia), then centrifuged (10 min, 100,000 × *g*) and resuspended in 900 μl of uncoating buffer (50 μg/ml). The uncoating reaction was allowed to reach steady-state for 10 min, 37 °C with full-length rat Hsc70 (1.3 μm) recombinantly produced in Sf9 cells, GST-auxilin(401–910) (bovine) (0.13 μm) recombinantly produced in BL21-DE bacteria, and ATP (0.5 mm) (36), then samples were centrifuged (30 min, 100,000 × *g*) to analyze pellet and supernatant by immunoblotting for CHCs.

### Immunogold labeling of HeLa CCVs

CCVs prepared from HeLa cells (Buffer A) were adsorbed to freshly glow-discharged formvar/carbon-coated copper mesh EM grids for 2 min. Samples were washed by transferring EM grids between 20-μl drops of buffer A to buffer G (25 mm HEPES, 125 mm potassium acetate, 5 mm magnesium acetate, pH 7.2), then fixed on 20 μl of 0.1% glutaraldehyde in buffer G for 15 min. Samples were blocked by transfer over buffer G, then PBS, then PBS with 0.15 m glycine, pH 7.2, for 5 min, and PBS with 1% (m/v) fish gelatin for 30 min. Samples were double-labeled with mouse monoclonal anti-CLC (decorating CHC17) (CON.1, 4 μg/ml) (33) and anti-CHC22 (SHL-KS, diluted 1:200) in PBS with 1% fish gelatin for 1 h, then washed by successive transfer over six 20-μl drops of PBS with 1% fish gelatin. To minimize background from secondary antibodies (5/10 nm gold-labeled goat anti-mouse/rabbit antibodies diluted 1:30 in 1% fish gelatin in PBS), diluted antibodies were pre-adsorbed on unlabeled, fixed HeLa CCVs on EM grids for 1 h, then 20 μl were used to detect primary antibody labeling for 1 h. After labeling, samples were washed three times in PBS with 1% fish gelatin, twice in PBS, then fixed in 2.5% glutaraldehyde in PBS (10 min), washed twice with PBS and distilled water and stained with (2% (m/v) uranyl acetate/water). All steps were at room temperature (22 °C).

### Statistical analysis of immunogold-labeled HeLa CCVs

Although only 6.9% of multi-labeled CCVs contained labels for CHC17 and CHC22, we asked if this proportion was significantly different from what we would expect if all CCV coats contained both heavy chains. This analysis is complicated by several factors, including: 1) the actual abundance of each protein in cells, CHC17 is ∼10 times more abundant than CHC22 (9); 2) the efficiency of each primary and secondary antibody used for staining, likely to be different for each epitope and difficult to estimate; and 3) the proportion of each CHC that is in CCVs as opposed to free in the cytosol, again unknown, although our previous data and those presented here indicate that CHC22 is more likely to be found on membranes than CHC17. Given these unknowns, we used the CCVs with only one label (by definition either CHC17 or CHC22) to empirically determine the likelihood of a given label being CHC17 or CHC22. This analysis showed that a given label had a 62.6% chance of being CHC17 and a 37.4% chance of being CHC22. We used these probabilities to calculate the expected number of dual-labeled CCVs. As this is simplest in the case of CCVs labeled with exactly two gold particles we limited our statistical analysis to this case, which included 190 analyzed CCVs. We calculated expected numbers to be: 1) both labels are CHC22 = 14 or 26% CCVs; 2) one CHC17 label and one CHC22 label = 47 or 89% CCVs; 3) both labels are CHC17 = 40 or 75% CCVs; if all CCVs are composed of CHC17 and CHC22 at a 10:1 ratio. We then conducted a χ^2^ test to determine whether our observed 11 dual-labeled CCVs was significantly lower than the expected 89 dual-labeled CCVs. This was highly significant (*p* < 0.0001, *n* = 190 CCVs with two labels, df = 1), suggesting that CHC17 and CHC22 form separate populations of CCVs.

### Embedding and ultra-thin sectioning of HeLa cells

For ultra-thin sectioning, siRNA-treated HeLa cells washed twice (5 mm EDTA, 4.5 g/liter of glucose in PBS, pH 7.3, 37 °C) were resuspended (same buffer, 10 min) and an aliquot was removed to assess expression of CHC isoforms by immunoblotting. Cells (pelleted 300 × *g*, 6 min, 4 °C) were fixed (3% glutaraldehyde in 0.1 m cacodylate, pH 7.2), then processed for embedding in Epon and stained as described (37). For quantification of clathrin-coated structures, images of ultra-thin sections of embedded cells were analyzed using NIH ImageJ by observers blind to condition.

### FACS-based assay of Tf uptake

HeLa cells were treated with siRNA targeting each CHC or All-Star Negative control siRNA (20 nm) (Qiagen) for 72 h. During the last 24 h of siRNA transfection, cDNA encoding green fluorescent protein (GFP)-tagged CHC17 or CHC22 was transfected and experiments were started the next day. For transferrin uptake assays, cells were starved of FBS for 2 h in uptake media (DMEM + 0.1% BSA) then incubated with Alexa Fluor 647-coupled transferrin (Tf-AF647 Thermo Fisher Scientific), 10 μg/ml in ice-cold uptake media; for 10 min on ice. Unbound Tf-AF647 was removed by 3 successive washes (ice-cold PBS). Uptake was then initiated by adding warm uptake media to the cells and endocytosis was allowed to proceed for 10 min at 37 °C. After uptake, cells were placed back on ice and residual surface-bound Tf-AF647 was removed by 3 successive washes (ice-cold PBS), followed by two acid washes (0.15 m glycine buffer, pH 3) to strip any remaining surface Tf-AF647, and finally a further 3 successive washes (ice-cold PBS). The cells were then fixed in 4% paraformaldehyde for 15 min on ice and then 15 min at room temperature. The cells were then gently lifted using a cell scraper, pelleted (800 × *g*, 8 min), and re-suspended in PBS, 2% BSA for subsequent flow cytometer analysis (LSRII, BD Biosciences) using the DIVA software (BD Biosciences) to simultaneously measure levels of cell-associated Tf-AF647 and levels of transfected CHC (GFP). Cells expressing GFP constructs were divided into three groups based on levels of expression (low, medium, and high) each representing one-third of the GFP fluorescence intensity spectrum.

To develop and validate the FACS-based Tf-uptake assay, three control conditions were evaluated for each assay: (i) cells were mock-treated without Tf-AF647 to determine the nonspecific fluorescent background; (ii) cells were fixed with paraformaldehyde after removal of excess Tf-AF647 to determine the amount of surface transferrin bound prior to uptake; (iii) cells were left on ice after binding Tf and then exposed to an acidic strip to test the efficacy of surface-bound Tf stripping. As removing surface Tf-AF647 with the acid wash was key for ensuring the specificity the assay, we verified for multiple repeats that condition (ii) was statistically different from conditions (i) and (iii) before proceeding with further assays (Fig. 5*A*) (one-way ANOVA, i *versus* ii, *p* < 0.0001; ii *versus* iii, *p* < 0.0001; i *versus* iii, NS). Cells were also immunoblotted to confirm CHC17 (Fig. 5, *C* and *D*) or CHC22 (*A* and *B*) depletion for each assay.

Flow cytometry data were analyzed using FlowJo (Treestar). Immunoblot samples were quantified (ImageJ, NIH) and normalized to their respective actin loading control. For each assay, FACS results were normalized to samples treated with control siRNA (siCon) with no CHC-expressing DNA, as 100% uptake. Results from six assays were pooled to create the graphs reported in Fig. 5. CHC22 expression, CHC17 expression, surface-bound Tf, and Tf-uptake were each analyzed by one-way ANOVA (GraphPad Prism) to determine the effect of CHC17 and CHC22 levels on Tf uptake.

## Author contributions

P. N. D., K. S., S. M. C., J. A. T., S. V., M. D. C., K. B., and A. R. performed experiments reported in the manuscript. Experiments were conceived by F. M. B., K. S., P. N. D., S. M. C., S. V., L. A. S., and C. J. S. and the manuscript was written by F. M. B., L. A. S., S. M. C., and P. N. D. with comments from other contributors.

## Supplementary Material

Supplemental Data

## Footnotes

4 The abbreviations used are:

CCV: clathrin-coated vesicle
CHC: clathrin heavy chain
CLC: clathrin light chain
GLUT4: glucose transporter 4
GSC: GLUT4 storage compartment
T2D: type 2 diabetes
TAE: Tris/acetate/EDTA
UC: uncoating complex
Tf: transferrin
ANOVA: analysis of variance.
