# Supplementary material for: CHC22 and CHC17 clathrins have distinct biochemical properties and display differential regulation and function
Source: J Biol Chem. 2017 Nov 2;292(51):20834–44. doi: 10.1074/jbc.M117.816256 (PMC5743061; doi:10.1074/jbc.M117.816256)
Supplement: Supplemental Data [file supp_292_51_20834__index.html]

CHC22 and CHC17 clathrins have distinct biochemical properties and display differential regulation and function — CHC22 and CHC17 clathrins have distinct biochemical properties and display differential regulation and function — CHC22 biochemistry — Supplemental Data 

# CHC22 and CHC17 clathrins have distinct biochemical properties and display differential regulation and function

## Supplemental Data

- Supplemental Figure 1 (.pdf, 1.2 MB) - Figure S1: Receptor-mediated endocytosis of transferrin is not affected by CHC22 depletion or overexpression.
