# Supplementary material for: CHC22 and CHC17 clathrins have distinct biochemical properties and display differential regulation and function
Source: J Biol Chem. 2017 Nov 2;292(51):20834–44. doi: 10.1074/jbc.M117.816256 (PMC5743061; doi:10.1074/jbc.M117.816256)
Supplement: Supplemental Data [file 10.1074_M117.816256_jbc.M117.816256-1.pdf]

Figure S1

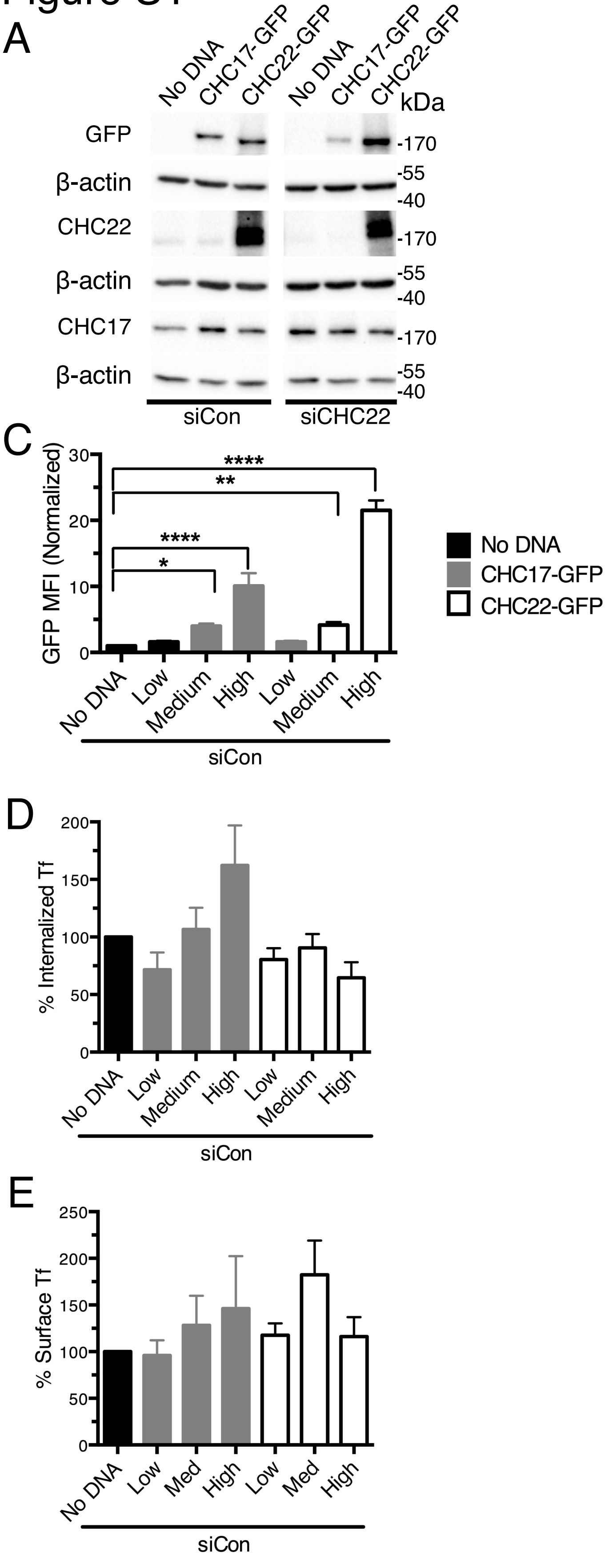

Receptor-mediated endocytosis of transferrin is not affected by CHC22 depletion or expression.

Figure S1: Receptor-mediated endocytosis of transferrin is not affected by CHC22 depletion or overexpression.
A. Representative immunoblot of endogenous CHC22, CHC17 and the expression of GFP-tagged CHC17 and CHC22 rescue constructs in HeLa cells transfected with control siRNA (siCon) or siRNA targeting CHC22 (siCHC22), used for experiments in (***C-E)*** and (***F-H***). For all blots, the migration positions of molecular mass markers are indicated at the right in kilodaltons (kDa). ***B.*** Quantification of CHC22 immunoblot signals from experiments analyzed in (***C-E)*** and (***F-H*)** (Student's t-test, control (siCon, no DNA) vs. siCHC22 (no DNA), * = P < 0.05, N = 5), normalized to the actin immunoblot signal for each sample. ***C.*** FACS-based quantification of GFP-CHC17 and GFP-CHC22 expression in HeLa cells after transfection with siCon and GFP-CHC17 or GFP-CHC22. The GFP-positive population of cells was divided in three groups with low, medium and high MFI of the transfected GFP-CHC for panels (***D)*** and (***E***) (One-way ANOVA, Control (no siRNA, no DNA) vs. all siCon conditions, * = P < 0.05, ** = P < 0.01, *** = P < 0.001, **** = P < 0.0001, N = 6). ***D.*** Tf uptake measured by FACS in transfected cells treated with siCon shown in **(C)**. Results were normalized to cells transfected with siCon, no DNA and expressed as percent internalized Tf (One-way ANOVA, control (no siRNA, no DNA) vs. all siCon conditions, not significant (NS); P > 0.05, N = 6). ***E.*** Surface-bound Tf-AF647 prior to Tf uptake measured by FACS for assays in (***C-E***) (One-way ANOVA, Control (no siRNA, no DNA) vs. all siCon conditions, NS; P > 0.05, N = 6). ***F.*** FACS-based quantification of GFP-CHC17 and GFP-CHC22 expression in HeLa cells after siCHC22 treatment and transfection of GFP-CHC17 or GFP-CHC22. The GFP-positive population of cells was divided in three groups with low, medium and high MFI for analysis in panels (***G*** and ***H***) (One-way ANOVA, Control (siCon, no DNA) vs. all siCHC22 conditions, **** = P < 0.0001, N = 6). ***G.*** Quantification of Tf uptake in transfected cells treated with siCHC22 as in (***F***). Results were normalized to cells treated with siCon (no DNA) and expressed as percent internalized Tf (One-way ANOVA, Control (siCon, no DNA) vs. all siCHC22 conditions, NS; P > 0.05, N = 6). ***H.*** Surface-bound Tf-AF647 prior to Tf uptake measured by FACS in cells treated with siCHC22, for assays in (***F-H***) (One-way ANOVA, control (siCon, no DNA) vs. all siCHC22 conditions, NS; P > 0.05).
